# Postoperative opioids, endocrine changes, and immunosuppression

**DOI:** 10.1097/PR9.0000000000000640

**Published:** 2018-02-23

**Authors:** Simon Haroutounian

**Affiliations:** Department of Anesthesiology and Washington University Pain Center, Washington University School of Medicine, St. Louis, MO, USA

## Abstract

Opioids are among most effective drugs for managing acute postoperative pain. This article discusses the potential effects of perioperative opioids on endocrine and immune function.

**Key Points**

1. Surgery is associated with a massive inflammatory and stress response. Untreated postoperative pain results in immunosuppression, increases the risk of thromboembolic events, and delays recovery.
2. Chronic opioid therapy results in major endocrine changes such as opioid-induced androgen deficiency and bone demineralization. The clinical relevance of these phenomena with short-term opioid use for postoperative pain is unclear.
3. In the surgical setting, high-dose opioids may contribute to inhibition of immune responses and curbing of stress response (eg, cortisol rise), but the clinical consequences of these observations are still unclear.
4. Multimodal postoperative approaches, especially those combining regional anesthesia with local anesthetics, help improve pain scores and reduce postoperative opioid requirements; however, contradictory data exist on their effect in reducing immune-mediated complications such as infections or tumor dissemination.

## 1. Introduction

“The pathetically grim and perspiring patient, fearful of moving or breathing, has become a constant fixture on postsurgical wards. His suffering was anticipated by his physician and is accepted in the knowledge that it will disappear in time.”^22^

Acute postoperative pain is considered an unavoidable consequence of a surgery, and is somewhat anticipated, given the (typically) massive tissue injury, inflammation, and stress response as a result of a surgical procedure. However, it is well documented that the “perspiring patient, who is fearful of moving or breathing,” ie, the patient who suffers from severe postoperative pain, is more prone to adverse outcomes. Patients, in whom severe pain impairs breathing, coughing, physical therapy, and early mobilization, are more likely to develop complications such as respiratory infections and thromboembolic events. The pain is also a significant stressor both from the psychological and physiological points of view.

The responses of the central nervous system and the hypothalamus-pituitary-adrenal (HPA) axis to a perceived stress involve a complex network of signaling molecules, including endorphins, catecholamines, and cortisol. The stress response to surgery is characterized by activation of HPA axis, as reflected by increased secretion of hypothalamic corticotropin-releasing hormone (CRH), and subsequently increased levels of adrenocorticotropic hormone (ACTH) and cortisol. In addition, surgical stress enhances the secretion of other catabolically active hormones, mainly catecholamines and glucagon, but also prolactin, growth hormone, and β-endorphin.^42^

The enhanced postsurgical stress response, which leads to high cortisol levels, can result in immunosuppression,^33^ supporting the notion that the relief of pain should be beneficial in preventing, eg, postoperative infections.^33^ In the setting of cancer surgery, the immune function may have even further implications, as animal studies have shown that surgery-induced stress is associated with impaired natural killer (NK) lymphocyte activity, impairing the body's ability to clear tumor cells.^33^

Although the description of a grim, perspiring, and suffering patient as a constant fixture may not have been surprising in the 1950s, one would expect that the development of novel drugs and multimodal analgesia approaches in the past few decades would dramatically improve the quality of postoperative pain management, and reduce the undesired hormonal and inflammatory consequences associated with the surgical stress. However, current data from inpatient and outpatient surgical settings indicate that between 30% and 50% of postoperative patients consistently experience moderate to severe pain.^19,35,36^

## 2. The role of opioids in the management of acute postoperative pain

Opioids are the most commonly used drugs for the management of acute postoperative pain. Depending on the setting and type of surgery, opioids are delivered systemically either through scheduled or pro re nata (as needed) dosing, or through a patient-controlled analgesia device. Alternatively, the postoperative opioid regimen may include neuraxial delivery through an epidural catheter, as a part of patient-controlled epidural analgesia (PCEA) technique. Mostly, opioids that are used for acute postoperative pain relief, include the short-acting morphine, hydromorphone, oxycodone (in countries where parenteral formulation is available), and occasionally fentanyl or tramadol (which is a weak opioid agonist and a serotonin–norepinephrine reuptake inhibitor).

Opioids are very effective in treating acute postoperative pain; however, not without important side effects. Among their most commonly observed and reported adverse effects in this setting are nausea, vomiting, sedation, pruritus, and constipation. The more severe undesired outcome is respiratory depression, which is potentially life-threatening, and is the most feared side effect of opioid medications. The brainstem control of respiratory rate and tidal volume is driven by afferent input of partial pressure of arterial O_2_ (through chemosensors in carotid and aortic bodies) and CO_2_ (through chemosensors in the brainstem). Opioids, through μ-opioid receptor-mediated depression of excitability of brainstem chemosensory neurons, depress the ventilatory response to increased CO_2_, thus depressing respiration.^31^

## 3. Literature search methodology and focus

This update will focus on a different subset of effects associated with the use of opioid analgesics in the acute postoperative setting; these are endocrine changes and immunosuppression. This clinical update will discuss the evidence behind the role of opioids in contributing to each of these phenomena in the postoperative setting, discuss their clinical relevance, and summarize the recent data on approaches that could be considered when treating pain in patients after surgery.

To provide systematicity to literature retrieval, PubMed search was performed in September 2017 with the following keywords: “opioids” (All Fields) AND “surgery” (All Fields) AND “pain” (All Fields) AND (“immune suppression” [All Fields] OR endocrine” [All Fields]). The search resulted in 125 articles, which were screened for relevant information, including articles identified from their reference lists.

To obtain a comprehensive evaluation of opioid effects in the perioperative setting, information on both postoperative opioids for pain relief and data on intraoperative opioids for analgesia/anesthesia purposes were considered. As rapid recovery from anesthesia is becoming an increasingly important outcome, opioids with faster offset such as remifentanil and sufentanil are used more commonly, but (especially remifentanil) may be associated with certain adverse effects.

## 4. Major endocrine changes associated with opioids

The current evidence suggests that opioids cause endocrine changes by 2 major mechanisms:

(1) Opioids affect the hypothalamic-pituitary-gonadal (HPG) axis.
(2) Opioids affect the HPA axis.

The impact on the HPG axis is a well-described dose-dependent effect of opioids, particularly related to treatment with daily doses above 100 to 200 mg of oral morphine equivalents for more than a few weeks. The HPG cascade is initiated by the release of gonadotropin-releasing hormone (GnRH) from the hypothalamus, which stimulates the anterior pituitary to release luteinizing hormone (LH) and follicle-stimulating hormone (FSH), which stimulate the gonads to produce testosterone and estrogen. Opioids bind to μ-opioid receptors in the hypothalamus, inhibiting the release of GnRH, thus decreasing LH and FSH secretion from the pituitary. Subsequently, this leads to decrease in testosterone levels and hypogonadism, a condition typically referred to as opioid-induced androgen deficiency (OPIAD).^29^

Opioid-induced androgen deficiency is a significant observation in the setting of chronic opioid therapy, affecting 53% to 90% of male patients on chronic opioid therapy.^29^ The long-term symptoms of OPIAD in this setting include decreased attention, decreased libido, fatigue, erectile dysfunction, and osteoporosis. In women receiving chronic opioids, LH and FSH levels are markedly reduced and are associated with amenorrhea and impaired adrenal androgen production.^13^

Some studies have questioned the suggestion that OPIAD is associated only with chronic opioid therapy. Several animal studies have shown that even acute opioid administration (eg, morphine, fentanyl, and buprenorphine), especially at high doses, results in reduced levels of testosterone,^10^ but the duration of this effect is reported to last between 24 hours to up to 8 weeks after treatment.^11^

A significant drop in total testosterone level in humans is also observed with a single ≥30 mg dose of methadone, or within 24 hours of perioperative morphine administration.^30,41^

Unfortunately, well-designed studies aimed at understanding the mechanisms of testosterone suppression, and the clinical relevance of short-term hypogonadism in the setting of acute opioid administration are lacking. Moreover, surgical stress may also contribute to these phenomena, and patients receiving ketamine (and not morphine) analgesia still display reduced testosterone plasma levels.^30^ Therefore, more careful studies are warranted to dissect the magnitude and the importance of OPIAD in the perioperative setting.

The impact of the opioids on the HPA axis is less well described. The HPA cascade is initiated by CRH release from the hypothalamus, which stimulates the pituitary to release ACTH to the systemic circulation, stimulating, in turn, the adrenal glands to produce 2 hormones—dehydroepiandrosterone (DHEA) and cortisol. Opioids seem to inhibit the functioning of the entire HPA axis. On one hand, they reduce the production/release of CRH, and on the other, they decrease the responsiveness of the anterior pituitary to CRH. Both processes lead to reduced ACTH secretion. Independently, opioids may also directly interfere with the adrenal gland production of cortisol and DHEA. Cortisol is important for mounting stress responses, and DHEA is an important precursor for testosterone (in men) and estradiol (in women).

The mechanistic data on opioid-associated changes in HPA axis come primarily from rodent and healthy volunteer experiments. For example, in healthy volunteers, single-dose morphine suppresses ACTH and cortisol levels both at baseline and after CRH stimulation.^2^ However, the extrapolation of healthy volunteer data to the surgical setting is not straightforward, as the surgical stress, and the postoperative pain per se, can have a substantial effect on the functionality of the HPA axis. The surgery typically results in an increased stress response, which subsides after about 24 hours. Certain opioids (remifentanil, particularly) are reported to acutely suppress plasma cortisol in a dose-dependent manner.^1^ In the setting of elective C-section, remifentanil administered as a bolus followed by continuous intraoperative infusion (compared with fentanyl administration after delivery) partially obtunded the neuroendocrine response to surgery with a decrease in ACTH rise (but not in norepinephrine, epinephrine, and growth hormone).^14^

The association between opioids and blunting ACTH or cortisol rise seems to be modest at best, and highly dependent on the timeframe of the assessment. In addition, some studies failed to find changes in cortisol levels after intraoperative remifentanil infusion.^3^ As in the case of reduced testosterone concentrations, the clinical relevance of altered cortisol levels in the setting of perioperative opioid therapy is not clear.

## 5. Opioids and immune response—inflammation and infections

Opioids do not possess strong anti-inflammatory properties such as nonsteroidal anti-inflammatory drugs or corticosteroid drugs, and their potential effects on inflammatory responses seem to be highly dependent on the setting. For example, in patients undergoing coronary artery bypass graft procedure, administration of continuous remifentanil infusions (vs intermittent fentanyl dosing) resulted in lower levels of proinflammatory cytokines (such as TNF-α and IL-6) at some time points after cardiopulmonary bypass. Eight hours after surgery, however, no differences were observed between the groups.^40^ On the contrary, some studies have reported lower inflammatory response (lower C-reactive protein level) with opioid-minimizing analgesia in the setting of colorectal surgery^38^ or enhanced proinflammatory cytokine release in the spinal cord as a response to opioid challenge. The clinical relevance of these short-term effects of opioids on inflammatory markers remains to be investigated.

The 2 main areas of research focused on opioid-associated immune effects deal with (1) the effects of opioids on immune response to infections; and (2) opioid effects on tumor-specific immune responses, affecting tumor growth and dissemination.

Major histocompatibility complex, class II (MHC-II) molecules, expressed on antigen-presenting cells, are important regulators of immune cell development and function. Morphine has been shown to alter gene expression of the MHC-II in circulating immunocytes (Beagles 2004), and thus suspected in causing immunosuppression. It is a matter of debate whether these effects are mediated by opioid-induced alteration in cortisol levels (as in adrenalectomized rats, morphine exposure does not affect MHC-II), or there is a direct immunosuppressive effect attributable to opioids. Interestingly, morphine withdrawal results in renewed increase in circulating corticosterone levels and a renewed suppression of MHC-II in previously opioid-tolerant animals.^28^

The risk of surgical site infection was higher after abdominal surgery with remifentanil anesthesia vs fentanyl anesthesia.^21^ The findings could be attributable to more substantial immunosuppression with remifentanil (although direct data are lacking), or to opioid withdrawal, which is more likely with the short-acting remifentanil. Additional data support this notion of immunosuppression associated with opioid withdrawal; for example, remifentanil discontinuation increased the risk of intensive care unit–acquired infection,^27^ and morphine withdrawal in mice (after 96-hour exposure) increased the risk of infection in an experimental model of septic shock. Interestingly, the most commonly detected organisms in tissue of morphine-withdrawn mice were bacteria that are part of the normal gastrointestinal flora.^18^ There are additional rodent studies suggesting that morphine, by altering gut microbiome, may increased the risk of sepsis by bacterial dissemination.^24^

A review examining whether opioids increase the risk of infections in the perioperative or intensive care setting, suggested that patients receiving higher doses of systemic opioids had an increased risk of developing pneumonia perioperatively.^32^ However, these results are not universal and were observed only if laparoscopic vs open surgery (or epidural vs systemic opioid therapy) was compared.

Overall, the data are both inconsistent and insufficient to determine the extent of opioid-associated immune suppression on infectious complications after surgery. Untreated pain itself can increase the risk of infections, eg, because of impaired mobility; pain also enhances the body's stress response, which by enhancing circulating cortisol, can contribute to immunosuppression. It needs to be taken into account that low-dose opioid control groups in some studies (eg, epidural analgesia and laparoscopic surgeries) could have better quality of analgesia and improved ability to clear secretions. It is also possible that increased risk of infection is among the immunological sequelae of opioid withdrawal, rather that opioid therapy, per se. The microbiome-related effects of opioids (and the potential dissemination of gut microorganisms) are another area that requires additional research. Although some studies suggest that buprenorphine (partial μ-opioid agonist and a κ-opioid antagonist) may be devoid of immunosuppressive effects,^33^ whether different opioids have differential effects on immune function still requires detailed investigation.

## 6. Opioids and immune response—cancer

Opioids can suppress NK cell cytotoxicity. Both high-dose and low-dose fentanyl suppressed NK cytotoxicity for 24 hours.^5^ However, rate of recovery of NK cell suppression was longer in the high-dose fentanyl group. Rats, which were treated with 20 mg/kg morphine (vs saline), developed a decrease in B-lymphocyte blood expression of MHC class II molecules within 2 hours.^4^ The same group has previously reported that in rats, fentanyl suppresses NK cell cytotoxicity and increases the risk of tumor metastases.^34^ Although there might be a dose-dependent effect, it is not clear whether different opioids affect the NK function differentially. In this regard, data suggest that endogenous opioids (β-endorphin) inhibit T-cell proliferation to a lesser extent than exogenous morphine.^15^

Despite these experimental findings, it is unclear whether there is any long-term immunosuppression associated with similar changes in antigen-presenting cells in humans.

A large retrospective analysis of a national registry data from Denmark (n = 34,188) showed no difference of breast cancer recurrence as a function of opioid use.^12^ The researchers categorized “strong immunosuppressive opioids (codeine, morphine, and fentanyl) vs weakly immunosuppressive opioids (oxycodone, tramadol, buprenorphine, and hydromorphone),” based on previous literature, but found no difference between the groups.

Considering the immunosuppressive effects attributable to pain and the enhanced stress response (and cortisol increase), which opioids may blunt, it is currently unclear whether the clinically relevant “net effect” of postoperative pain management with opioids tips the immune balance towards immune suppression. There is also insufficient evidence to determine that some opioids produce strong immunosuppressive effects, whereas others produce only weak or no such effects.

## 7. Mitigation approaches

### 7.1. Stress response and inflammation

Inflammatory reaction after surgery is a physiological response that helps the healing process. An excessive inflammatory response can lead to complications, but immune suppression could negatively affect the healing process. With potential effects of opioids on inflammation, and potential immunosuppressant activity, the attempt in the recent years has been to use multimodal analgesia approaches that provide adequate analgesia, but avoid excessive opioid use, especially high-dose intraoperative remifentanil.

Patient-controlled epidural analgesia (with bupivacaine and fentanyl) after lower abdominal surgery have resulted in blunted postoperative elevation of cortisol and prolactin, and lower pain scores, compared with other opioid-only systemic analgesia regimens.^42^

In a setting of retropubic prostatectomy, PCEA (with ropivacaine and sufentanil) resulted in lower pain intensity, and reduced the postoperative stress response (plasma cortisol and glucose), but not the acute inflammatory response (TNF-α and IL-6 levels).^17^

Another study in 24 women undergoing laparoscopic cholecystectomy assessed the effect of a single-dose intrathecal (bupivacaine with fentanyl) vs epidural (ropivacaine with fentanyl) anesthesia, before general anesthesia in both groups.^7^ Intraoperative cortisol, noradrenaline, and total catecholamine levels were significantly lower in the intrathecal (spinal) anesthesia group (and patients required less systemic fentanyl); ie, spinal anaesthesia produced a more favourable endocrine response than epidural anaesthesia.

In pediatric cardiac surgery, on the other hand, high-dose intraoperative fentanyl has demonstrated better (lower) profile of stress markers such as ACTH, glucose, cortisol, and lactate, compared with low-dose fentanyl. High-dose intraoperative fentanyl also resulted in lower postoperative opioid requirements.^26^

The available results demonstrate that neuraxial anesthesia and analgesia (intrathecal route perhaps more advantageous than epidural), result in either lower pain scores or less systemic opioid requirements, and can blunt the stress response, with minimal effect on acute inflammatory response. This suggests that multimodal anesthesia with neuraxial opioid and local anesthetic delivery, where possible, may provide advantage to systemic (especially high-dose) opioid administration. The advantages in the pediatric surgery setting have not been explored sufficiently and merit more thorough investigation.

### 7.2. Cancer

The literature is divided on the topic of regional anesthesia approaches and their potential effect on cancer-related outcomes. Initial retrospective studies reported that perioperative use of regional (neuraxial or peripheral) anesthesia is associated with improved outcomes in terms of cancer recurrence and survival after breast and prostate surgery.^6,16^

However, a later retrospective study in colorectal cancer surgery,^20^ an ad hoc analysis of a prostatectomy study,^37^ and a retrospective study in lung cancer did not show any benefit of regional anesthesia on cancer-related outcomes.^9^ A prospective randomized controlled trial^25^ did not demonstrate prolonged survival in major abdominal surgery, and a recent retrospective study in patients undergoing radical cystectomy for bladder cancer demonstrated more than 50% reduction in perioperative opioid consumption with spinal anesthesia, but this difference was not associated with changes in outcomes such as all-cause mortality, bladder cancer mortality, or cancer recurrence.^39^

A prospective study^8^ found that innate immunity (NK cells, CD4^+^, and CD8^+^ cells) was depressed in lung cancer patients undergoing resection, but postoperative epidural analgesia did not help preserve the immunity.

In addition, a recent systematic review of the literature (15 studies) found inconclusive evidence to support or refute the suggestion that paravertebral blocks in breast cancer can reduce cancer recurrence or improve survival.

Currently, there is no conclusive evidence that regional anesthesia, by either reducing opioid doses or by other mechanisms such as sympathetic blockade, can improve long-term cancer-related outcomes in cancer patients undergoing surgery. With that said, there is no major disadvantage in using regional techniques, and the opioid-sparing and stress response–blunting acute effects justify the widespread use of such approaches for postoperative pain relief.

## 8. Critical questions to be addressed by future research

The undesired effects attributable to opioids, which were discussed in this article, are often challenging to address, as some of these effects may be related to surgical stress or postoperative pain, per se. For example, endogenous opioids such as beta-endorphins play an important role in the regulation of gonadotropins through modulation of GnRH pulse amplitude and frequency. Therefore, with the question of OPIAD in mind, it is critical to control for this variable, as some data suggest that testosterone levels are lower in subjects with pain compared with controls, irrespective of opioid use.^23^ Are then patients, who are in more pain (and therefore are likely to require higher opioid dose) more likely to develop endocrine adverse effects? What is then the contribution of pain vs opioid (type and dose)? Is it more important to control the pain well, or avoid high-dose opioids, even at the expense of higher pain? These research questions are yet to be answered and need to be address accurately to move the field forward, toward safer and more rational perioperative opioid use.

In a similar scenario, severe pain is associated with enhanced stress response, accompanied by catecholamine and cortisol release. Numerous preclinical and clinical studies have focused on the effect of opioid analgesics on the postoperative stress response. As enhanced stress response may cause immune suppression and lead to postsurgical complications, blunting the stress response has been historically considered as a desirable perioperative outcome. However, the advantages of blunting the stress response with opioids should be weighed against potential immunosuppression, and more research is needed to achieve an optimal balance that would positively affect patient outcomes.

## Disclosures

The author has no conflict of interest to declare.
